# Sense of coherence is associated with reduced psychological responses to job stressors among Japanese factory workers

**DOI:** 10.1186/1756-0500-5-247

**Published:** 2012-07-06

**Authors:** Kayoko Urakawa, Kazuhito Yokoyama, Hiroaki Itoh

**Affiliations:** 1 Department of Stress Science, Mie University Graduate School of Medicine, 2-174 Edobashi, Tsu, Mie, 514-8507, Japan; 2 Department of Epidemiology and Environmental Health, Juntendo University Faculty of Medicine, 2-1-1 Hongo, Bunkyo-ku, Tokyo, 113-8421, Japan

**Keywords:** Sense of coherence, Job stress, Brief Job Stress Questionnaire, Stress reduction

## Abstract

**Background:**

Job stress is associated with adverse health effects. The present study was conducted to examine the association between sense of coherence (SOC), as advocated by Antonovsky, and psychological responses to job stressors among Japanese workers.

**Methods:**

A self-administered questionnaire containing a Japanese version of the 13-item SOC scale, the Brief Job Stress Questionnaire, and a self-rated health item were distributed to 1968 workers in X Prefecture. Anonymous responses were recovered by postal mail.

**Results:**

Complete responses were received from 299 workers (response rate 15.2%, 191 males and 108 females) who consented to participate in the study. Participants were 186 office clerks, 38 sales representatives, 22 technical engineers, 16 service trade workers, eight information processing workers, eight technical experts, and 21 other workers of various types. SOC scores were associated with age, self-rated health, job title, and marriage status. According to regression analyses stratified by gender, SOC was inversely associated with tension, fatigue, anxiety, depression and subjective symptoms in males, and tension, depression and subjective symptoms in females. SOC was positively associated with vigor in both males and females.

**Conclusions:**

Having a strong SOC may reduce worker’s negative job stress responses and increase their vigor. Longitudinal studies are required to confirm this finding.

## Background

Approximately 30 years have passed since Aaron Antonovsky [1] proposed the salutogenic model of health. The sense of coherence (SOC) concept, which is based on the salutogenic model, has attracted research attention in the population health field. One’s SOC is believed to express the extent to which they have a persistent, enduring but dynamic feeling of confidence that: (1) the stimuli deriving from their internal and external environments in the course of living are structured, predictable, and explicable (comprehensibility); (2) resources are available to them to meet the demands posed by these stimuli (manageability); and (3) such demands are challenges, worthy of investment and engagement (meaningfulness) [1-3]. SOC in individuals is assessed by a 13-item scale [4].

Several studies have indicated that SOC modifies of job stress responses. For example, having a strong SOC protects individuals from the effects of stressors at work [5]. Health social workers with a strong SOC experience less burnout than those with a weak SOC [6]. SOC buffers the effects of stressful life events on individuals’ mental health status [7]. A study of Japanese civil servants indicated that having a stronger SOC was associated with fewer days of absence from work and fewer adverse physical health symptoms [8]. Among resident physicians, an increase in psychiatric complaints as measured by General Health Questionnaire (GHQ) scores was associated with a weak SOC and adverse work conditions as assessed by the Brief Job Stress Questionnaire (BJSQ) [9].

Developed in Japan for use in occupational health check-ups, the BJSQ assesses job stressors and stress responses [10]. The BJSQ is a multi-dimensional questionnaire using four-point Likert scale response options (1 “strongly disagree” to 4 “strongly agree”) to measure job stressors and psychological distress such as tension, fatigue, anxiety, depression, and vigor (three items for each) and subjective symptoms (10 items). Quantitative job demand, qualitative job demand, job decision latitude (three items for each) and social support (nine items) are the job stressors measured by the BJSQ, which is essentially based on the demand–control–support model [11,12] (in addition, physical job load is measured by one item). All of the BJSQ scales have acceptable levels of internal consistency, reliability and factor-based validity [13,14].

Our previous study [15] demonstrated that GHQ scores were positively associated with job demands as measured by the BJSQ, whereas the scores were inversely associated with SOC scores among Japanese workers in the manufacturing industry, suggesting that having a stronger SOC can reduce the adverse effects of job stress such as psychiatric complaints. The purpose of the present study was to test this hypothesis by examining the association between SOC and the stress responses assessed by the BJSQ. In addition, one item evaluated self-rated health as a stress response, because it is reported to be a sensitive indicator of health status among community residents [16].

## Methods

Study participants were employees of 50 small enterprises (2–49 employees) in X Prefecture, Japan. We asked a representative from each company to distribute self-administered questionnaires containing a Japanese version of the SOC scale [1], the BJSQ [10], the self-rated health item and socio-demographic items to their employees. A total of 1968 workers received the questionnaire. The purpose of the study was explained to the workers by their supervisors as being “to investigate the relationships between mental health status and job stress”. They were instructed to anonymously complete the questionnaire and return it by postal mail. Of the 1968 workers invited to participate, 299 returned the questionnaire (15.2%, 191 males and 108 females). They consisted of 186 office clerks, 38 sales representatives, 22 technical engineers, 16 service trade workers, eight information processing workers, eight technical experts, and 21 other workers of various types. The SOC scale [1] consisted of 13 items assessing comprehensibility, manageability and meaningfulness. Participants were asked to rate each item (1–7 points), and the points were summed into an SOC score. They were also asked to complete the BJSQ. Self-rated health was evaluated by asking participants to rate their own health status (1 “very healthy”, 2 “rather healthy”, 3 “not so healthy”, 4 “unhealthy”); the rating was reversed for use in further analysis.

Associations between SOC and BJSQ scores, and self-rated health and socio-demographic variables were analyzed by U-tests, Chi-square tests and stepwise multiple regression using SPSS version 11.0 for Windows. In the multiple regression analysis, stress responses evaluated by the BJSQ and self-rated health scores were the dependent variables, and age, duration of work, managerial work (yes = 1, no = 0), marriage (married = 1, not married = 0), SOC scores, and job stressors assessed by the BJSQ were the independent variables. In the first models (model 1), all independent variables were entered into (*p* ≤ 0.05) or removed from (*p* > 0.05) the regression equations based on the significance level of the partial regression coefficient. In the second models (model 2), age, duration of work, managerial work, and marriage were entered into the equations as possible confounders, and then the stepwise method was used for the remaining variables.

Because gender differences have been found in the effects of SOC on health status [7,17] and in the association between job stress and health behaviors [18], the analyses were performed separately for each gender.

The study design was reviewed and approved by the Research Ethics Committee of Mie University Graduate School of Medicine.

## Results

The 299 participants had a mean age of 49.3 years (SD = 11.4). The mean SOC score was 58.8 (SD = 14.0) with a Cronbach’s alpha coefficient of 0.863. Male participants were significantly older than female participants (mean age 50.7 versus 46.4 years, respectively, *p* < 0.05). Males had significantly higher SOC scores (mean = 60.4, SD = 13.7) than females (mean = 56.7, SD = 13.6, *p* < 0.01). There were no significant differences in BJSQ or self-rated health scores between the genders.

Participants were divided into two groups based on their SOC scores (above and below average, high and low SOC groups, respectively). Socio-demographic variables, BJSQ and self-rated health scores for each group are presented in Table 1. Participants in the high SOC group were older, more of them were married, and there were more managerial workers compared with the low SOC group. Job stressors (low support) and stress responses were lower in the high SOC group, while self-rated health scores were higher (indicating that participants in this group considered themselves healthier).

**Table 1:** **Differences in socio-demographic variables, self-rated health and BJSQ scores between SOC groups**^**a**^

	Higher SOC	Lower SOC	Differences (p)^b^
	Mean (standard deviation)		
Age (years)	52.2 (11.1)	45.6(10.5)	<0.001
BJSQ (scores)			
*Stressors*			
Job demand (quantity)^c^	6.8(2.4)	6.3(2.3)	ns
Job demand (quality) ^c^	6.6(2.1)	6.3(2.0)	ns
Low job latitude	5.3(1.5)	6.5(1.8)	<0.001
Low support (supervisor)	6.1(2.0)	7.4(2.1)	<0.001
Low support (coworkers)	6.5(1.9)	7.4(2.1)	<0.001
Low support (friends and family)			
Physical job load ^c^	3.2(0.9)	3.2 (0.8)	ns
*Stress responses*			
Tension	5.5(2.0)	7.6(2.6)	<0.001
Fatigue	5.3(1.9)	6.9(2.3)	<0.001
Anxiety	5.4(1.8)	6.7(2.0)	<0.001
Depression	8.7(2.6)	11.9(4.2)	<0.001
Subjective symptoms	16.7(5.2)	19.4(5.1)	<0.001
Vigor	8.3(2.2)	6.1(2.1)	<0.001
Self-rated health(scores)	2.1(0.5)	1.9(0.4)	<0.01
	Number (%)		
Managerial workers	119 (62%)	72 (38%)	<0.001
Married	139(55%)	113(45%)	<0.05

^a^ SOC scores of 59 or above and 58 or less (n = 151 and 146, respectively).

^b^*U*-test or chi-square test.

^c^ Reversed item (the higher the score, the lower the demand or load).

Results of the multiple regression analyses are shown in Tables 2 and 3 for males, and Tables 4 and 5 for females. In models with and without controlling for socio-demographic variables, SOC was inversely associated with stress responses, i.e. tension, fatigue, anxiety, depression, and subjective symptoms, and was positively associated with vigor. Stress responses and self-rated health were adversely associated with job stressors such as job demand, low support and job aptitude in males. Similarly, SOC was inversely associated with tension, depression, and subjective symptoms, and was positively associated with vigor. Stress responses were adversely associated with job stressors such as job demand and low support in females. Marriage was inversely associated with depression in males, whereas it was positively associated with anxiety and depression in females. The correlation coefficients for the associations between SOC and the stress responses are presented in Table 6.

**Table 2:** **Associations of stress responses and self-rated health with SOC and stressors in 190 male workers: model 1**^**a**^

***Dependent variables***	Adjusted R^2^	Independent variables (partial regression coefficient) ^b^
***Tension***	0.214***	SOC (−0.408***)
		Job demand (quantity) (−0.193**)
***Fatigue***	0.306**	SOC (−0.390***)
		Job demand (quantity) (−0.357***)
***Anxiety***	0.313***	SOC (−0.352***)
		Job demand (quality) (−0.349***)
		Low support from supervisor (0.187*)
***Depression***	0.335***	SOC (−0.411***)
		Low support from supervisor (0.197**)
		Married (−0.195**)
		Job demand (quantity) (−0.158*)
***Subjective symptoms***	0.092***	SOC (−0.314***)
***Vigor***	0.395***	SOC (0.275**)
		Low job aptitude (−0.206**)
		Low support from supervisor (−0.254***)
***Self-rated health***	0.018*	Physical job load (−0.183**)
		Work duration (0.182*)
		Low support from colleagues (−0.157*)

^a^ See Methods section for the stepwise multiple regression model procedure.

^b^ Only those significantly associated with dependent variables are shown.

*p < 0.05 **p < 0.01 ***p < 0.001.

**Table 3:** **Associations of stress responses and self-rated health with SOC and stressors in 190 male workers: model 2**^**a**^

***Dependent variables***	Adjusted R^2^	Independent variables (partial regression coefficient) ^b^
***Tension***	0.231***	Managerial work (0.201*)
		SOC (−0.415***)
		Job demand (quantity) (−0.162*)
***Fatigue***	0.318***	Age (−0.185*)
		SOC (−0.391***)
		Job demand (quantity) (−0.321***)
***Anxiety***	0.300***	SOC (−0.360***)
		Job demand (quality) (−0.332***)
		Low support from supervisor (0.180*)
***Depression***	0.333***	Married (−0.212**)
		SOC (−0.428***)
		Low support from supervisor (0.188**)
		Job demand (quantity) (−0.158*)
***Subjective symptoms***	0.117***	SOC (−0.381***)
***Vigor***	0.395***	Low support from supervisor (−0.269***)
		SOC (0.254**)
		Physical job load (−0.194**)
		Low job aptitude (−0.195*)
***Self-rated health***	−	−

^a^ See Methods section for the stepwise multiple regression model procedure.

^b^ Only those significantly associated with dependent variables are shown.

*p < 0.05 **p < 0.01 ***p < 0.001.

**Table 4:** **Associations of stress responses and self-rated health with SOC and stressors in 101 female workers: model 1**^**a**^

***Dependent variables***	Adjusted R^2^	Independent variables (partial regression coefficient) ^b^
***Tension***	0.350***	SOC (−0.490***)
		Job demand (quantity) (−0.321**)
***Fatigue***	0.228***	Job demand (quantity) (−0.488***)
***Anxiety***	0.370***	Job demand (quantity) (−0.438***)
		Age(0.321***)
		SOC(−0.336**)
		Married (0.192*)
***Depression***	0.351***	Low support from supervisor (0.267*)
		Job demand (quantity) (−0.283**)
		SOC (−0.355**)
		Work duration (0.260**)
		Married (0.195*)
***Subjective symptoms***	0.123**	SOC (−0.264*)
		Low support from friends and family (0.236*)
***Vigor***	0.259***	SOC (0.409***)
		Low support from friends and family (−0.268***)
***Self-rated health***	−	−

^a^ See Methods section for the stepwise multiple regression model procedure.

^b^ Only those significantly associated with dependent variables are shown.

*p < 0.05 **p < 0.01 ***p < 0.001.

**Table 5:** **Associations of stress responses and self-rated health with SOC and stressors in 101 female workers: model 2**^**a**^

***Dependent variables***	Adjusted R^2^	Independent variables (partial regression coefficient) ^b^
***Tension***	0.345***	SOC (−0.540***)
		Job demand (quantity) (−0.326**)
***Fatigue***	0.215***	Job demand (quantity) (−0.511***)
***Anxiety***	0.370***	Age (0.338*)
		Married (0.206*)
		Job demand (quantity) (−0.435***)
		SOC (−0.336**)
***Depression***	0.345***	SOC (−0.370**)
		Job demand (quantity) (−0.280**)
		Low support from supervisor (0.270*)
***Subjective symptoms***	0.113**	SOC (−0.353**)
***Vigor***	0.258***	SOC (0.458***)
		Low support from friends and family (−0.257*)
***Self-rated health***	−	−

^a^ See Methods section for the stepwise multiple regression model procedure.

^b^ Only those significantly associated with dependent variables are shown.

*p < 0.05 **p < 0.01 ***p < 0.001.

**Table 6:** Correlation coefficients for SOC and stressors in 190 male and 101 female workers

	Males	Females
***Tension***	−0.425***	−0.525***
***Fatigue***	−0.460**	−0.236*
***Anxiety***	−0.436***	−0.245*
***Depression***	−0.504**	−0.452***
***Subjective symptoms***	−0.324***	−0.391**
***Vigor***	0.503**	0.481***
***Self-rated health***	0.243**	0.081

*p < 0.05 **p < 0.01 ***p < 0.001.

## Discussion

### Results of the multiple regression analysis

We found that among both male and female workers, stress responses such as tension, fatigue, depression, and anxiety, as well as positive mood (vigor) were inversely associated with job stressors as measured by the BJSQ. SOC had the opposite association with stress responses, indicating that having a stronger SOC can reduce the adverse effects of job stress on psychological status among workers of both genders. Thus, not only psychiatric complaints as measured by the GHQ, but also job stress responses are affected by one’s SOC.

Interestingly, marriage was positively associated with anxiety and depression in female workers, but inversely associated with depression in male workers. This is in contrast with a recent study conducted over 23 European countries on gender differences in depression, which demonstrated that a lower risk of depression was associated with marriage for both genders [19]. Marriage-related psychological distress may be greater in Japanese women than men. It was reported that among infertile Japanese women, having a lack of husband support was associated with increased depression and anxiety [20].

According to a previous review, there is a strong association between SOC and mental health [21]. For example, because SOC is inversely associated with anxiety, hopelessness, depression, and perceived stressors, and positively associated with optimism, hardiness, self-esteem, self-efficacy, and social skills, it would appear that people with a stronger SOC tend to positively view their health. The narrower SOC concepts, i.e. comprehensibility (how to understand reality) and manageability (ability to manage without great difficulty), may affect one’s perception of stress. It is suggested that reducing negative job stress responses by supporting workers’ self-perceptions as well as increasing their SOC and vigor can help them maintain a healthy life.

Eriksson et al. [22] demonstrated that SOC was significantly related to the self-rated health of 1013 Finland residents. We did not identify this association in our regression analyses, although the high SOC group had higher self-rated health scores than the low SOC group. This inconsistency between the studies may have resulted from differences in participant characteristics, and/or the smaller number of participants in our study. However, because self-rated health can predict one’s ability to cope with stress to some extent, self-rated health is verified as a simple and effective indicator that can be used in health screening to predict changes in future health conditions [16].

### Characteristics of high SOC workers

In our study, having a stronger SOC was associated with older age and job title (Table 1), although the groups were based on the mean SOC score, rather than a validated cut-off point. This association may be attributable to a number of causes. First, older people are more likely to be appointed to managerial positions. Second, people who are promoted to management positions will develop stress tolerance with experience [23]. Third, people who have a consistently good mental health status and can manage stress tend to be selected for management positions [24]. The positive association of SOC with older age and job title seems reasonable if SOC is not an inborn or innate capacity but a learned sense that is acquired a posteriori through various life experiences and becomes deeply ingrained to shape personality [25].

The positive association between marriage and SOC identified in the present study may also be attributable to the age of married people. According to Eriksson and Lindström [26], SOC tends to increase with age through the whole life span. Whether the increases in individual SOC are an effect of natural selection of people (healthy people survive) or a question of people developing a strong SOC staying well is not clear. They suggest the second explanation. Males usually have a stronger SOC than females, although the differences are small [26]. This agrees with the observation of a stronger SOC in males than in females in the present study.

### Significance of SOC at workplace

Horita [27] reported that SOC scores were significantly higher in healthy workers who had a high level of satisfaction with their work and life. According to a review of SOC and quality of life (QOL) [28], SOC seems to be a resource that directly enhances one’s QOL, or enhances it via good health perceptions. A stronger SOC and increased job control resulted in better subjective feelings of well-being in a 1-year follow-up study of Japanese civil servants [29]. On the other hand, a weak SOC is assumed to have either a direct relationship with poor psychological well-being or an indirect relationship via the development of lifestyle problems such as a lack of exercise, obesity or smoking [30]. These findings, together with the “antagonistic” effects of having a weak SOC on responses to job stressors demonstrated in the present study, indicate that enhancing workers’ SOC is essential for health at work and in daily life. Health education programs can improve workers’ SOC [31], and therefore worksite intervention programs should be developed in further studies.

### Study limitations

The present study was based on relatively small number of participants with a low response rate. This may have distorted the findings because the participants may have been more concerned about mental illness in relation to job stress than non-participants. Although it has been reported that the SOC and BJSQ scales have good reliability and/or validity [13,14,26], it remains to be elucidated whether the different scales used in the present study really measured the same underlying psychological constructs. Our findings may have been dependent on the participants’ general satisfaction or mood status, as the study was cross-sectional and used subjective-report variables. We only examined stress-related outcomes. In future studies, the prevalence/incidence of somatic or psychological disorders should be used as outcomes in relation to SOC and the stressors measured by the BJSQ; because, for example, coronary heart disease risk is significantly related to job stress [11,12]. A well-designed longitudinal study following a larger number of participants is necessary to further confirm that SOC reduces the adverse health effects of job stress.

## Conclusion

It is suggested that increasing one’s SOC may reduce their negative job stress responses and subjective symptoms, and enhance their vigor. The positive association of SOC with older age and job title indicates that SOC is not an inborn or innate capacity but a learned sense. Interventions for increasing workers’ SOC should be developed.

## Misc

Kayoko Urakawa and Kazuhito Yokoyama contributed equally to this work.

## Competing interests

The authors declare that they have no competing interests.

## Authors’ contributions

KU and KY equally contributed to the study design, data collection, analysis, interpretation, and manuscript preparation. HI helped with manuscript preparation as well as data analysis and interpretation. All authors read and approved the final manuscript.
